# Bottom Temperature Effect on Growth of Multiple Demersal Fish Species in Flemish Cap, Northwest Atlantic

**DOI:** 10.3390/ani15081120

**Published:** 2025-04-12

**Authors:** Krerkkrai Songin, Fran Saborido-Rey, Graham J. Pierce

**Affiliations:** Instituto de Investigaciones Marinas (IIM), Consejo Superior de Investigaciones Científicas (CSIC), 36208 Vigo, Spain; fran@iim.csic.es (F.S.-R.); g.j.pierce@iim.csic.es (G.J.P.)

**Keywords:** climate change, GAMM, VBGF, asymptotic length, marine ecology

## Abstract

This study investigates the effects of warming water on growth in seven demersal fish species including Atlantic cod (*Gadus morhua*), American plaice (*Hippoglossoides platessoides*), Greenland halibut (*Reinhardtius hippoglossoides*), roughhead grenadier (*Macrourus berglax*) and three species of redfish (*Sebastes* spp.) in the Northwest Atlantic and compares the changes in growth across species. Length-at-age data were collected from EU bottom trawl surveys from 1993 to 2018, and bottom temperature data were obtained from the Copernicus Marine Service. Generalised additive mixed models (GAMMs) were used to describe the temperature effects on growth. The analysis was carried out separately for males and females. Both sexes of all species except American plaice showed significant temperature effects on growth. To obtain the growth parameters, von Bertalanffy growth functions (VBGFs) were fitted to the predictions from best-fit GAMMs for all species and both sexes under five different bottom temperature scenarios (3, 3.5, 4, 4.5 and 5 °C). The predictions from all best-fit GAMMs were broadly similar in form to the fitted von Bertalanffy growth functions (R^2^ > 90%). Increased bottom temperature generally resulted in a decrease in the asymptotic length (*L_∞_*) and an increase in the growth rate (*k*). The species with the most dramatic increase in *k* over the temperature range of 3 °C to 5 °C was Atlantic cod, for which *k* increased from 0.05 to 0.13 year^−1^ in females and from 0.08 to 0.14 year^−1^ in males. The maximum length (*L_max_*), predicted by the VBGF at maximum age generally declined from 3 °C to 5 °C. The species with the most pronounced decline in *L_max_* was beaked redfish (*S. mentella*). An increase in the proportion of smaller individuals could impact population productivity and result in lower biomass available to fisheries. Uneven changes in fish growth in the warming ocean could also have wider ecological implications and alter the trophic landscape.

## Simple Summary

This study examines temperature effects on the growth of seven demersal fish species in Flemish Cap. These species are Atlantic cod, American plaice, Greenland halibut, roughhead grenadier and three species of redfish. The length-at-age and bottom temperature data were analysed using generalised additive mixed models (GAMMs). The analysis was carried out separately for males and females. Both sexes of all species except American plaice showed significant temperature effects on growth. Best-fit GAMMs were used to predict the length at age under five temperature scenarios (3 °C, 3.5 °C, 4 °C, 4.5 °C and 5 °C). Von Bertalanffy growth functions were then fitted to the predictions to estimate asymptotic length and growth rate. As the temperature increased, most species showed an increase in growth rate but a decline in size at later life stages. Atlantic cod experienced the largest rise in growth rate, particularly in females. Beaked redfish showed the most drastic decline in size at maximum age. These findings suggest that warming oceans may lead to smaller fish, which could reduce the overall biomass available to fisheries. Differences in growth changes across species could also disrupt marine ecosystems and alter predator–prey relationships. Understanding these effects is important for fishery management and ecosystem conservation in the changing climate.

## 1. Introduction

Global oceans have experienced rapid environmental changes, especially increased temperature of both near-surface and deep ocean waters [1]. The temperature in the Northwest Atlantic is projected to increase by nearly three times the global average [2,3]. These changes pose a threat to temperature-sensitive species, potentially impeding the recovery of already depleted populations, as observed in the demersal community of the southern Gulf of St. Lawrence and Flemish Cap Atlantic cod (*Gadus morhua*) [4,5].

Water temperature has a profound influence on fish life history, as it can directly alter fish metabolism and key parameters such as growth, maturity and mortality [6,7,8]. The impact of temperature changes on fish growth has been extensively documented globally, even in deep-sea environments (beyond 200 m depth), which are often regarded as stable [9]. A recent study has shown that temperature can impact the growth of a deep-sea scorpion fish species differently in different habitats [10]. This suggests that temperature impacts on growth should be investigated locally when possible, as the results for one species and location may not be applicable to similar species in other locations.

Alterations in life history parameters can have implications far beyond the individual level, influencing both population productivity and trophic structure [11]. While environmental changes can be detrimental to some species, they may pose less threat to (or even favour) others, depending on their resilience, which can lead to ecosystem-level changes [12]. Such environmental impacts may also affect fisheries by reducing (or increasing) stock biomass and maximum sustainable yield [13]. The magnitude of these impacts on an ecosystem is indicative of its resilience, where resilience at ecosystem-level can be defined as “the extent to which ecosystems can absorb recurrent natural and human perturbations and continue to regenerate without slowly degrading or unexpectedly flipping into alternate states” [14]. To comprehend the potential ramifications of global change for ecosystems and fisheries, it is crucial to examine the temperature effects on life history across a broad range of species and trophic levels [15].

One of the important life history aspects that could be affected by the temperature changes is growth. Knowledge of growth is necessary for estimating a fishing mortality benchmark that corresponds to the maximum sustainable yield [16]. A review of over a thousand publications found that the climate change effects on fish growth were generally negative, primarily resulting in a reduction in growth [15]. The changes seen in growth were also found to relate to other aspects of life history including reproduction and lifespan [17,18]. Therefore, understanding the effects of climate change on growth in fish stocks is an important step towards understanding the wider array of its impacts.

Flemish Cap presents a valuable opportunity to study the influence of temporal and spatial variation in environmental factors on fish populations, thanks to extensive fishery-independent survey data collected by the EU over three decades [19]. This area harbours multiple commercially important fish species, including Atlantic cod, redfish (*Sebastes* spp.), Greenland halibut (*Reinhardtius hippoglossoides*), American plaice (*Hippoglossoides platessoides*) and roughhead grenadier (*Macrourus berglax*) [20]. These species exhibit diverse behaviours, diets, habitat preferences and reproductive strategies, which may result in varied growth responses to environmental changes [21,22,23]. A recent study showed that Atlantic cod growth in the area has decreased over the years due to a combination of changes in temperature, fishing pressure and food availability, especially at early ages [5], with the significance of each factor varying depending on the fishing pressure (e.g., the moratorium period versus periods of fishing activity).

The temperature at Flemish Cap exhibits significant spatial and temporal variation, including directional change, with a discernible increasing trend since the early 1990s [24,25]. This makes the area ideal for studying the effects of long-term temperature fluctuations on high-priority populations. The findings could also be relevant for similar ecological regions where data collection has been less intensive and extensive.

This study aims to evaluate the influence of temperature variations on growth in multiple demersal species, employing generalised additive mixed modelling and the traditional von Bertalanffy growth function. The analysis was performed separately for males and females due to sex-related growth differences that have been observed in redfish, Greenland halibut, witch flounder and roughhead grenadier in Flemish Cap [26,27,28,29]. This study also aims to forecast growth trends separately for both sexes of each species under different climate scenarios. Thus, we hope to provide insights into the sensitivity of multiple fish species to warming seas.

## 2. Material and Methods

### 2.1. Fish Sampling and Aging

Bottom trawl survey data from Flemish Cap, collected by the EU between 1993 and 2018, were utilized in this study. The surveys were conducted annually during the summer months (late June to early August) by four collaborating institutes: the Institute of Marine Research (IIM-CSIC), the Spanish Institute of Oceanography (IEO), the AZTI-Tecnalia Foundation and the Portuguese Institute for Sea and Atmosphere (IPMA). The sampling methodology is described in detail in a publication by the Northwest Atlantic Fisheries Organization [30].

**Figure 1:**
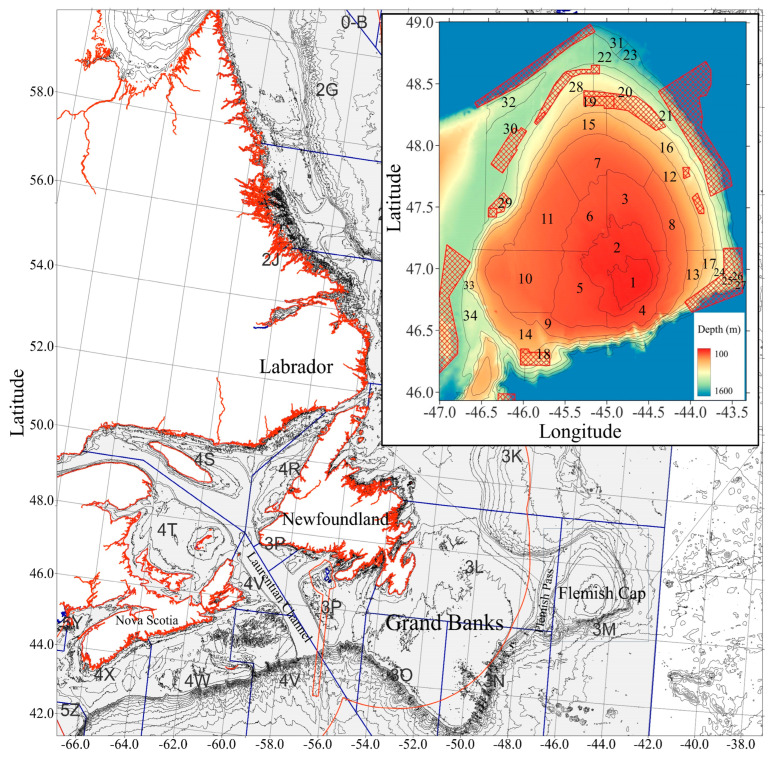
Flemish Cap depth and sampling strata and NAFO vulnerable marine ecosystem closure areas (red mesh). The Canadian exclusive economic zone is indicated by the red border. NAFO division boundaries are represented by the blue border. Original figure from Songin et al. 2024 [33].

The fish samples were collected using the RV Cornide de Saavedra (1993–2002) and the RV Vizconde de Eza (2003 onwards). The study area was divided into 34 strata for stratified random sampling (Figure 1). The initial 19 strata were limited to a depth of ≤730 m and were consistently sampled in all years. The additional 15 strata (730–1460 m depth) were sampled from 2004 onward, except for strata 26 to 27, which were sampled only during 2004–2007; subsequently, sampling was discontinued due to the presence of dense sponge growth. These areas were considered to belong to vulnerable marine ecosystems, in which bottom fishing activities were then prohibited [31,32].

Fish samples were identified at the species level, except for juvenile redfish (fork length < 15 cm), for which identification at the species level was not practical on board. Seven species were selected for the study due to their common occurrence and commercial importance: Atlantic cod, American plaice, Greenland halibut, roughhead grenadier, Acadian redfish (*S. fasciatus*), beaked redfish (*S. mentella*) and golden redfish (*S. norvegicus*). Biological data, including length, weight, sex and maturity stage, and sagittal otoliths, were collected from these species. The length measurement method varied according to the species due to their different morphological characteristic. Total length (TL) was measured for Atlantic cod, American plaice and Greenland halibut, while fork length (FL) was recorded for redfish, and anal length (AL) for roughhead grenadier. The target for biological sampling was to collect sagittal otoliths from 20 individuals from each 1 cm length class for both males and females, every year [30]. Age determination was performed by otolith reading in laboratories by technicians at the IIM and IOCC.

### 2.2. Temperature Data

**Figure 2:**
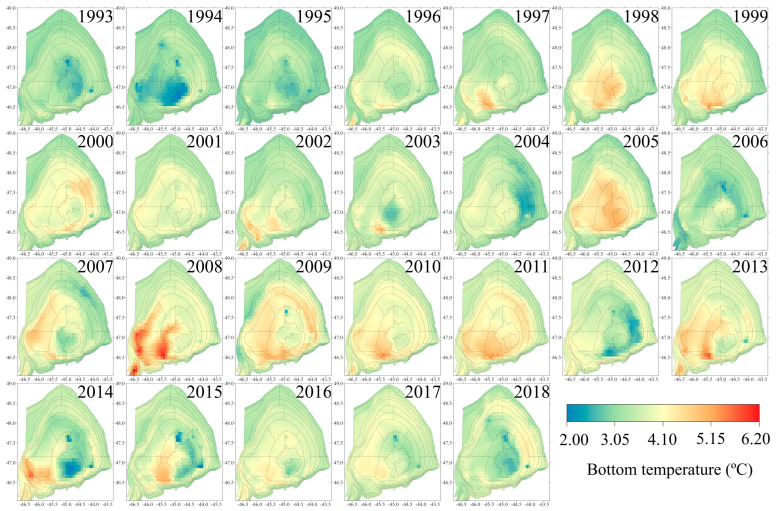
Maps of average July Flemish Cap bottom temperature in 1993–2018 extracted from data provided by Copernicus Marine Service.

July monthly average bottom temperatures (BTs) for each year from 1993 to 2018 were obtained from the Copernicus Marine Service (CMEMS). The monthly average temperature was used instead of the in situ data to minimise the contribution of day-to-day BT variation within each survey. The BT data were based on reanalyses from the CMEMS global ocean reanalysis products, which incorporate real-time global forecasting [25]. The standard grids used had a spatial resolution of 1/12° (Figure 2). Temperature values were attributed to trawls based on the specific grids corresponding to the sampling locations.

### 2.3. Length-At-Age Models

Gaussian generalised additive mixed models (GAMM) were employed to analyse the non-linear relationship between length and the independent variables age and bottom temperature (BT) for both sexes of all selected species. Haul (identification) numbers were included as a random variable to account for the likely higher similarity of individual fish within hauls. The models were developed using RStudio version 4.1.1 with the mgcv package [34]. The possible models can be described as follows:

Model with interaction between age and BT:

(1) $$L=s\left(Age,\;BT\right)+haul\;ID$$

Model without the interaction term:

(2) $$L=s\left(Age\right)+s(BT)+haul$$

Model with only age as a predictor:

(3) L=s(Age)+haul

where *L* represents the length, and *s* denotes the regression spline fitted to a predictor. The spline used for the modelling was the “thin-plate regression spline”, chosen for its flexibility and data-driven aspect, given the absence of prior knowledge of the forms of relationships among the variables. The restricted maximum likelihood (REML) method was used to estimate the smoothness parameters of the smooth functions in GAMMs. The adequacy of the basis dimension (k) was checked for all best-fit GAMMs using the gam.check function. The model likelihood values were compared using the Akaike information criterion (AIC) difference. The best-fit model was determined based on the lowest AIC, considering that between-model differences in AIC (ΔAIC) are considered significant only if the models differ by more than two AIC units and that if the models have a similar AIC value, according to the principle of parsimony, the simplest model is preferred. The best-fit model was used to predict length at age under five different bottom temperatures (3, 3.5, 4, 4.5 and 5 °C), which are within the spatiotemporal temperature range seen in Flemish Cap.

Von Bertalanffy growth functions (VBGFs) were fitted to the predictions from the best-fit GAMMs for each species. The VBGF is given by:

(4) $$L_{t}=L_{\infty }(1-e^{-k\left(t-t_{0}\right)})$$

where *L_t_* is the length at age *t*, *L_∞_* is the asymptotic length, *k* is the growth coefficient, and *t*_0_ is the age at which the length is 0. The best-fit VBGF was determined for each combination of species and sex under all temperature scenarios based on the minimum standard deviation. The maximum age (*t_max_*) used to fit the VBGF was estimated as the average age of the ten oldest samples to avoid including outliers in the calculation of the growth parameters.

The potential maximum length (*L_max_*) that a fish can achieve within its natural lifespan was estimated by predicting the length at *t_max_* for each species and sex. To quantify the magnitude of variation, the differences in *L_max_* under the 3.5–5 °C temperature scenarios from *L_max_* at 3 °C were calculated using the following formula:

(5) $${\Delta L}_{3\;{}^{\circ}C-i\;{}^{\circ}C}=(1-\frac{L_{max,\;i{}^{\circ}C}}{L_{max,\;3\;{}^{\circ}C}})\times 100$$

where ${\Delta L}_{3\;{}^{\circ}C-i{}^{\circ}C}$ represents the length difference, and *L_max,i °C_* denotes the maximum length in a given *i* temperature scenario.

## 3. Results

The number of otoliths collected for age reading varied over time and across species during the period from 1993 to 2018. Among the species, Atlantic cod required the highest level of effort for age determination. The age data represented 25 surveys (i.e., 25 years), and in total, more than 10,000 otoliths were read for each sex (Table 1). Other species, including Greenland halibut, American plaice, roughhead grenadier and beaked redfish, also had age data from over 20 surveys. Although fewer otoliths were read for Acadian and golden redfish, the age data still represented more than 10 surveys.

### 3.1. Maximum Ages

The overall lifespan *t_max_* was calculated for all species (Table 2). Beaked redfish had the longest lifespan, followed by golden redfish, roughhead grenadier, American plaice, Acadian redfish, Greenland halibut and Atlantic cod. *t_max_* was higher for females than for males in all species except beaked redfish. The species with the largest differences in *t_max_* between the sexes were roughhead grenadier (7.9 years) and Greenland halibut (6.9 years). The species with the smallest differences in *t_max_* between males and females were Atlantic cod (1.8 years) and golden redfish (1.8 years). For all species except beaked redfish, the *t_max_* calculated from the data set with combined sexes was very close to, or exactly the same as, the female *t_max_*. This is because the ten oldest individuals were mostly or entirely female. On the other hand, for beaked redfish, the *t_max_* for combined sexes was close to the male *t_max_* because most of the ten oldest individuals were male.

**Figure 3:**
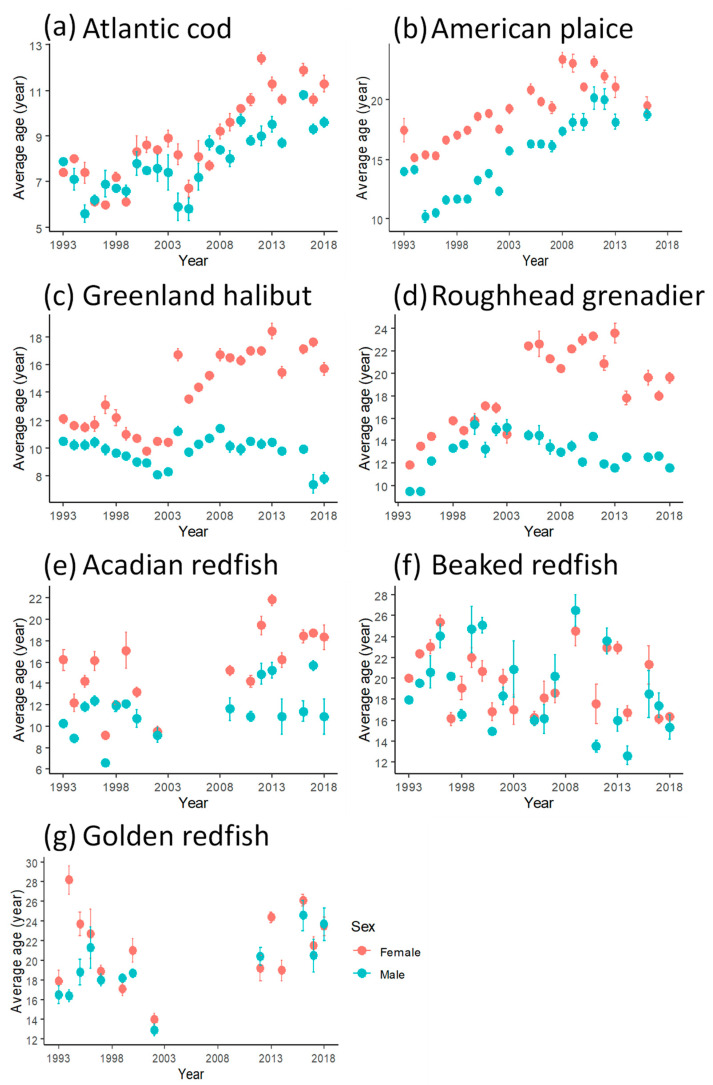
Average age of the ten oldest individuals (per calendar year) assessed by otolith reading in seven demersal fish species in Flemish Cap from 1993 to 2018. Note that the x-axis scale varies for display purposes.

The annual *t_max_* for years with otolith reading data showed temporal variations (Figure 3). For Atlantic cod and American plaice, the annual *t_max_* showed a clear increasing trend with time. A decrease in *t_max_* over time was seen for beaked redfish. For Acadian and golden redfish, there were no age data between 2003 and 2011, and no discernible temporal trend for *t_max_* could be detected.

For Greenland halibut and roughhead grenadier, there was a notable increase in female *t_max_* in 2004, coinciding with the expansion of the trawl sampling area from the limit of 730 m depth to that of 1460 m. In males of these species, *t_max_* was more stable over the years, although temporal trends could still be observed. The *t_max_* of male Greenland halibut declined steadily from 1993 to 2003. A shift to a higher *t_max_* in 2004 was seen in male Greenland halibut, but the change was much less marked than in females. The male *t_max_* then remained relatively stable until 2017, when it dropped substantially. The *t_max_* of both male and female roughhead grenadier clearly increased between 1993 and 2003. The male *t_max_* declined steadily after 2004.

**Figure 4:**
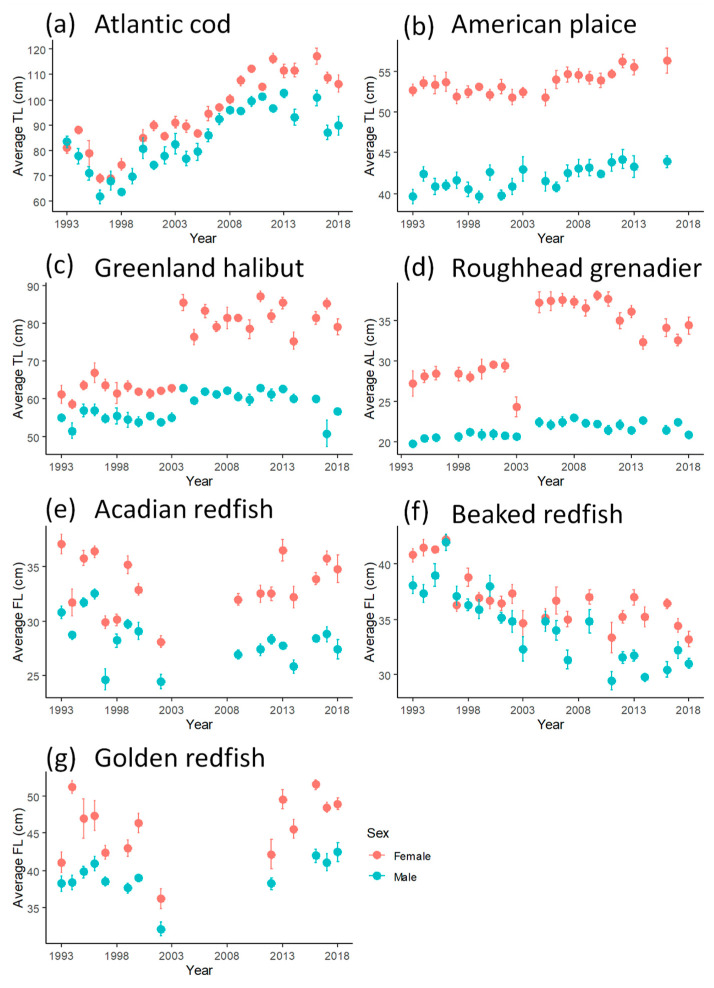
Average size of the ten oldest individuals (per calendar year) assessed by otolith reading for seven demersal fish species in Flemish Cap from 1993 to 2018. Note that the x-axis scale varies for display purposes.

The average size of the ten oldest individuals in each year was also calculated (Figure 4). The trends for size at oldest age for both sexes of Atlantic cod showed a steady increase since 1996, similar to the trends for *t_max_*. For American plaice, trends of increasing size were present but much less distinct than the increase of *t_max_* over the years. On the other hand, for beaked redfish, the decline in size was much more discernible than the trend for *t_max_*. The trends for size for the other two redfish species remained unclear, as was the case for their annual *t_max_*. For Greenland halibut and roughhead grenadier, a shift to a larger size in 2004, especially for females, could also be clearly observed in these data.

### 3.2. Temperature Coverage

**Figure 5:**
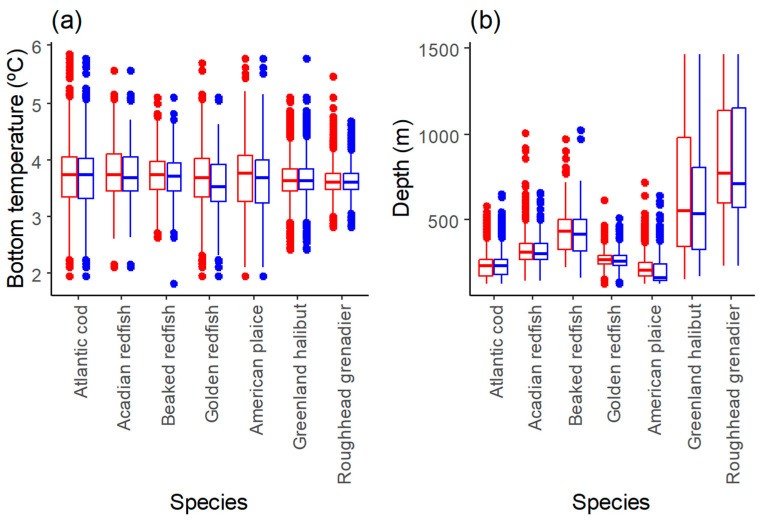
Boxplot depicting (**a**) the monthly average bottom temperature in July and (**b**) the depth at the sampling locations for female (red) and male (blue) individuals of seven demersal fish species in Flemish Cap from 1993 to 2018.

Species typically sampled at shallower depths were found at a wider range of temperature compared to those found at greater depths (Figure 5). Among them, Atlantic cod, golden redfish and American plaice experienced the widest temperature range at sampling locations (Figure 5a), with interquartile ranges spanning from 3.2 to 4.1 °C. The minimum recorded temperature was approximately 1.9 °C, while the maximum approached 5.9 °C. These three species were found at relatively shallow depths compared to others (Figure 5b), with a median sampling depth of <270 m, and a third quartile depth of <300 m. Acadian and beaked redfish, which inhabited deeper areas with a median depth of <420 m, displayed a slightly narrower interquartile temperature range.

In contrast, the species with the narrowest temperature ranges were Greenland halibut and roughhead grenadier, with interquartile ranges between 3.5 and 3.8 °C (Figure 5a). These two species occupied the deepest parts of underwater mountain compared to all others in this study (Figure 5b). The median sampling depth for Greenland halibut was 540 m for males and 554 m for females. Roughhead grenadier was found even deeper, with median depths of 709 m for males and 766 m for females.

### 3.3. Length-at-Age GAMM

The length-at-age models for each species were developed using GAMMs. The deviance explained exceeded 80% in all models (Table 3). The BT term was found to be highly significant (*p* < 0.001) when incorporated into the models for all species except American plaice and golden redfish. Apart from American plaice, most of the best-fit models featured a significant interaction between the effects of age and BT (*p* < 0.01), including for golden redfish, which did not show a significant BT term without an interaction. Only the best-fit GAMMs for roughhead grenadier of both sexes had a BT term without an interaction.

**Figure 6:**
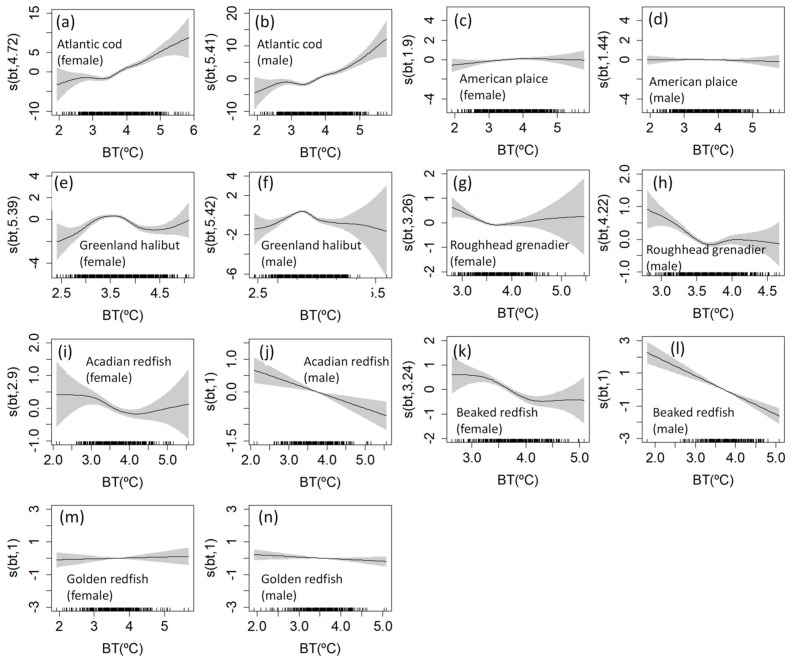
Gaussian GAMM smoothers for the partial effect of bottom temperature on size for seven demersal fish species, by sex. The subfigures (**a**–**n**) depict each combination of species and sex. The shaded areas represent 95% confidence intervals, with each mark along the inside of the x-axis corresponding to a single observation (the “rugplot”). Note that the scale of the x and y-axes varies among plots for display purposes.

The relationships between temperature and size (not accounting for the interaction with age) were explored, and differences in trajectories were found among the species. Clear differences were also apparent between the sexes for some species. The partial effects of temperature on size are shown in Figure 5. For Atlantic cod, the size increased with temperature for both sexes, especially at temperatures warmer than 3.5 °C, although the 95% confidence interval (CI) rapidly widened when the temperature rose above 4.5 °C (Figure 6a,b). Wide CIs were generally seen for model fits at temperatures below 3 °C and higher than 4.5 °C for all species and sexes. For American plaice, for which the temperature term was not significant in the best-fit GAMM, the size effectively remained unchanged regardless of the temperature (Figure 6c,d). For Greenland halibut, the size increased when the temperature increased from 2.5 °C to around 3.5 °C, then declined at higher temperatures up to 4.5 °C (Figure 6e,f). However, the trends for each sex diverged as the temperature continued to increase. The size of females increased again while the size of males continued its decline. For both sexes of roughhead grenadier, the size decreased when the temperature increased from 3 °C to around 3.5 °C and then remained relatively stable at warmer temperatures (Figure 6g,h). The size–temperature relationship for Acadian and beaked redfish was generally similar. For males of both species, the relationship was negative and linear (Figure 6j,l). However, the scale of the temperature effect was larger for beaked redfish. For females of both Acadian and beaked redfish, the size remained stable up to around 3 °C before it declined until the temperature reached 4 °C (Figure 6i,k). The size then slightly increased at warmer temperatures for Acadian redfish, while it remained fairly stable for beaked redfish. Although the interaction term between age and temperature was significant in the best-fit GAMM for golden redfish, the temperature term alone was not significant and did not affect the size (Figure 6m,n).

**Figure 7:**
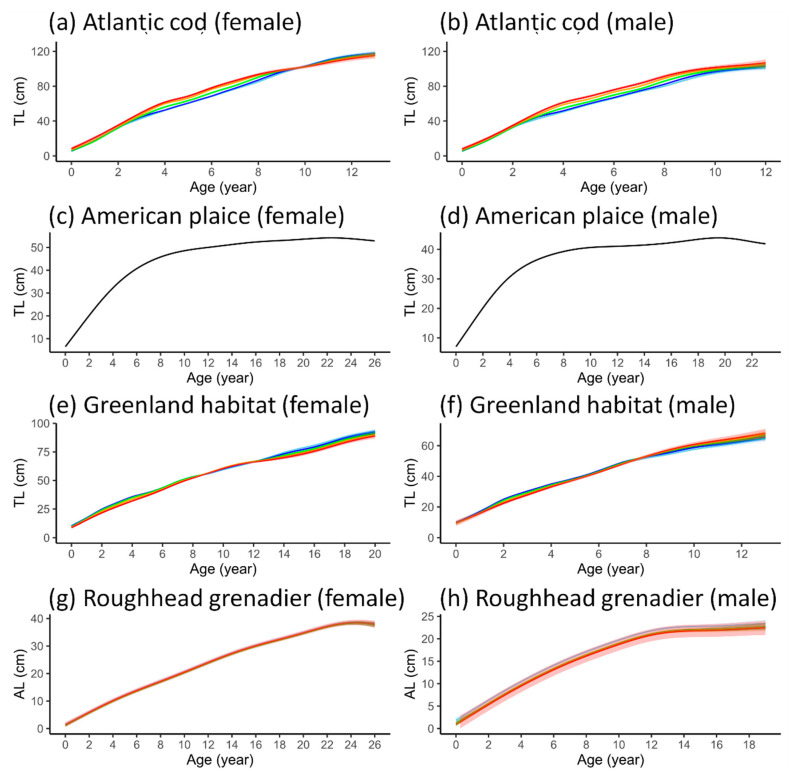

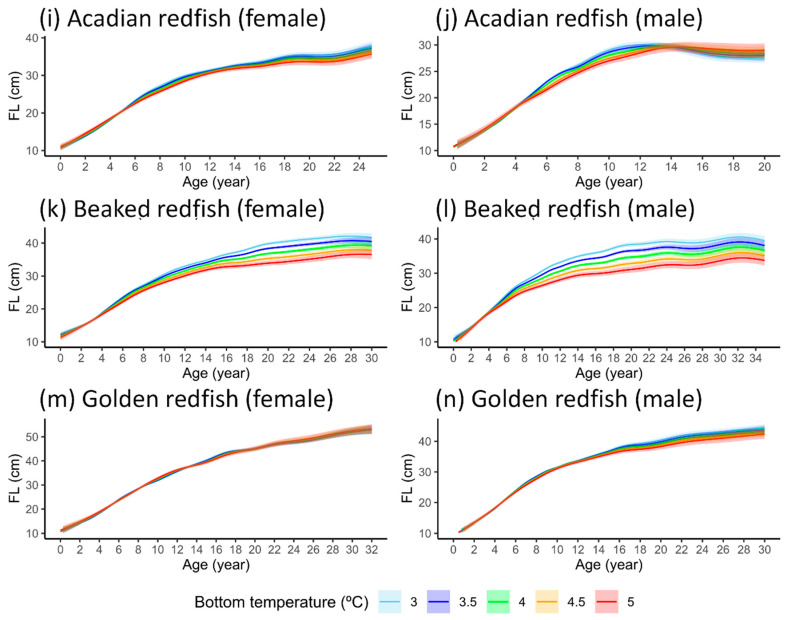
Growth trajectories in five average bottom temperature scenarios as predicted by best-fit GAMMs, with 95% confidence intervals. The different bottom temperatures are represented by different colours. For American plaice, the best-fit models do not include the temperature variable, and the trajectories are presented in black.

Although the best-fit GAMMs featured a significant BT effect or a significant effect of age × BT (i.e., the term capturing both main effects and their interaction) for most species, the magnitude of the temperature effects on growth differed from one species to another (Figure 7). The GAMM predictions at smaller increases in temperature for all species and sexes except American plaice can be found in Supplementary Materials (Figure S1). For both sexes of Atlantic cod, the models showed larger sizes at early life (<6 years) at higher temperatures (Figure 7a,b). However, female cod reached a larger final size in colder BT, while male cod grew larger in warmer BT. The GAMM predictions for length at age at 3 °C to 5 °C BT for both sexes showed the most pronounced size variation between the ages of 4 and 8 years. Within this range, the CIs for the length trajectories at 3 °C and 3.5 °C almost entirely overlapped. The sizes at 4 °C were consistently larger than at 3 °C and 3.5 °C but smaller than at 4.5 °C and 5 °C throughout the age range. The sizes at 4 °C were smaller than at 5 °C, but the 95% CIs indicated some overlap between them. After the age of 8 years, the differences in length at age at different temperatures began to decrease. Female cod became slightly larger in colder water after the age of 10 years, while male cod remained slightly larger in warmer temperatures. However, the widening CIs at ages >10 years led to overlaps in size at different temperatures.

In the early life stages (ages 2 to 4 years) of both male and female Greenland halibut, the size was slightly larger at lower temperatures (Figure 7e,f). After the age of 4 years, the length-at-age trajectories converged and continued to overlap through to later life stages, when the curves of the two sexes began to diverge. For female Greenland halibut, the temperature effects became more pronounced after the age of 12 years, with size increasing at colder temperatures (Figure 7e). the model predictions for the ages of 12 to 18 years at five different temperatures showed some overlap between the sizes at 3 °C and 3.5 °C and between the sizes at 4.5 °C and 5 °C. In contrast, for male Greenland halibut, the temperature effect indicated that the fish reached a larger size at warmer temperatures (Figure 7f). Size differentiation in males began around the age of 8 years, although large portions of the 95% CIs overlapped across different temperatures.

Although the best-fit GAMMs for both female and male roughhead grenadier contained a significant BT term, the temperature effect on the age–length relationship was virtually imperceptible in females (Figure 7g). For males, the size was larger at lower temperatures (Figure 7h). However, the model predictions showed overlapping length-at-age 95% CIs across all temperatures for both sexes.

For Acadian redfish, both males and females exhibited larger sizes at lower temperatures between the ages of 5 and 12 years (Figure 7i,j). the model predictions within this age range displayed a greater magnitude of temperature-driven differences in males than in females. The length trajectories then diverged at older ages. Female fish continued to be larger at lower temperatures throughout their lifespan (Figure 7i), while male fish became larger as they approached the final age (Figure 7j). However, the length 95% CIs after the age of 12 years were especially wide for male, and overlaps occurred across all temperatures.

Both sexes of beaked redfish exhibited similar temperature effects on the age–length relationship (Figure 7k,l). The model predictions showed that the growth trajectories at different BTs began to diverge after the age of 4 years, when the length at age at low temperatures became greater. The trend of a larger size at lower temperatures continued consistently throughout their lifespan. The 95% CIs widened and started to show some overlaps only when the fish reached their late 20s.

Golden redfish exhibited smaller temperature effects on growth compared to other redfish species (Figure 7m,n). The growth variation related to BT in females was almost entirely imperceptible, while males showed slightly smaller sizes in late life stages at higher temperatures. In females, the model predictions showed overlapping 95% CIs between the growth trajectories at all five temperatures for almost their entire lifespan, except between the ages of 14 and 18 years, when the sizes at low temperatures were slightly larger (Figure 7m). In males, the sizes at low temperatures became distinctly larger at the age of 16 years and older (Figure 7n), although the 95% CIs greatly widened after the age of 22 years, making the differences in growth difficult to determine.

### 3.4. VBGF Parameterization

**Figure 8:**
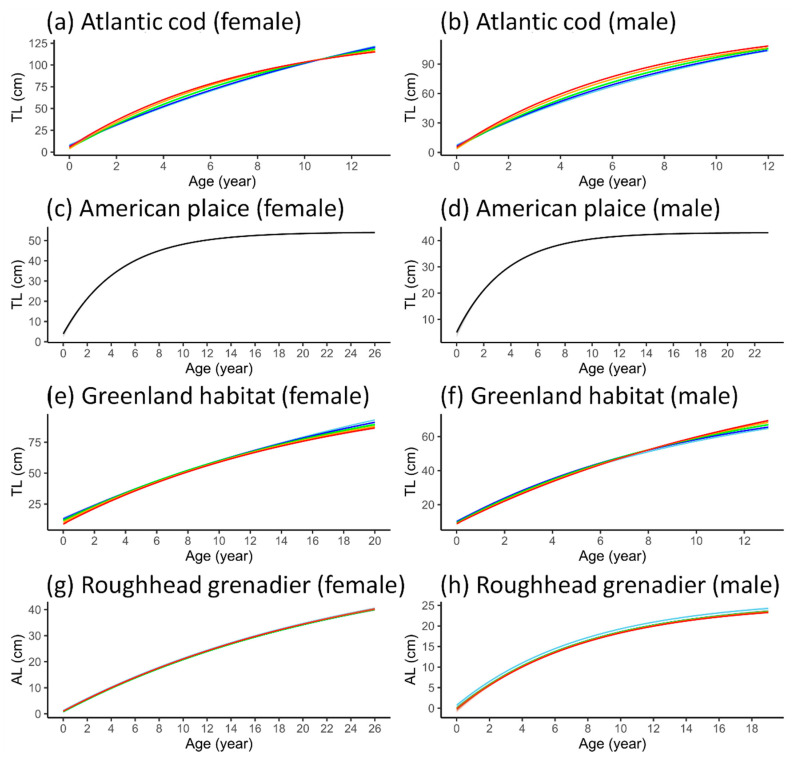

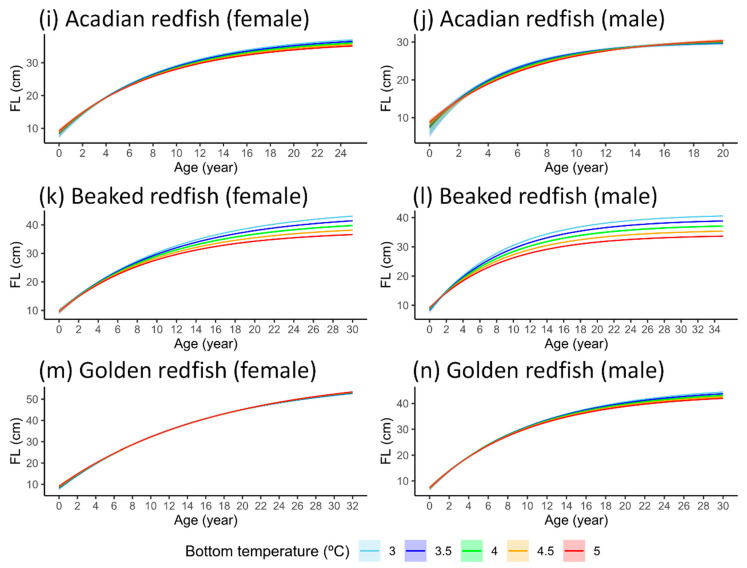
Growth trajectories in five average bottom temperatures scenarios as predicted by the von Bertalanffy growth function, with 95% confidence intervals. The different bottom temperatures are represented by different colours. For American plaice, the best-fit models do not include the temperature variable, and the trajectories are presented in black.

Apart from American plaice, for which the best GAMMs did not include BT, the VBGF could be fitted to the predictions of length at age generated by the best-fit GAMMs under five BT scenarios (R^2^ > 0.95). The growth trajectories from VBGF also visually resembled those of the GAMMs (Figure 8). The parameters derived from the fitted VBGF exhibited variation at different temperatures for all species, albeit to differing extents (Table 4). Females of all species exhibited lower *k* values but larger *L_∞_* values compared to males. The VBGF fitted to the predictions from the best-fit GAMMs for roughhead grenadier, which lacked an interaction term, showed no changes in *k* across the different temperature scenarios. The differences in *L_∞_* were also minuscule, with an increase of <1 cm from 3 °C to 5 °C. Most species exhibited a decreasing trend for *L_∞_* and increasing *k* as BT rose, regardless of sex. However, male Greenland halibut, male Acadian redfish and female golden redfish displayed opposing trends, although the largest difference in *k* for female golden redfish was less than 0.01.

**Figure 9:**
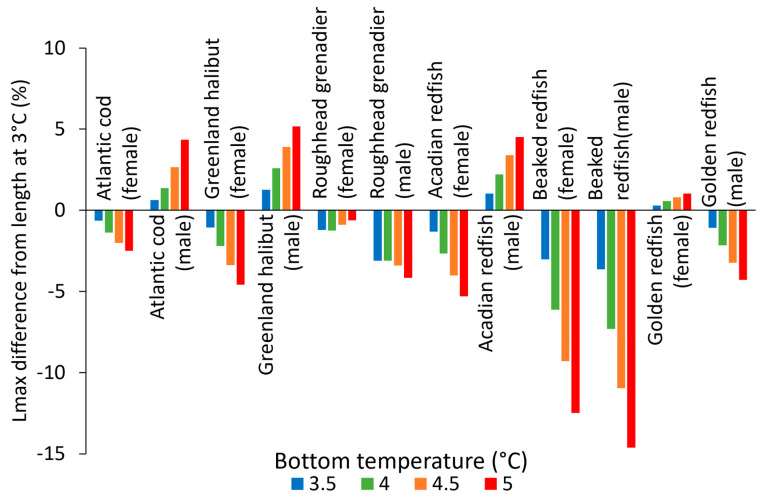
Differences in length at maximum age (*L_max_*) in four temperature scenarios from that in the 3 °C scenario, by species and sex.

Most species–sex combinations displayed differences in *L_max_*, mostly of less than 5%, when the temperature increased from 3 to 5 °C (Figure 9). The species–sex combinations that showed differences exceeding 5% were male Greenland halibut, female Acadian redfish and, most notably, beaked redfish of both sexes. The species–sex combinations with the least *L_max_* variation were female roughhead grenadier and female Acadian redfish, with the greatest *L_max_* variation being around 1% when the temperature changed from 3 to 5 °C. The case of male Atlantic cod is notable because *L_∞_* decreased at higher temperature but *L_max_* showed the opposite trend. This was due to the greatly increased *k* at higher temperatures and the relatively low *t_max_*. The higher *k* allowed male cod in warmer scenarios to grow much more rapidly in their early life, while the low *t_max_* prevented it from reaching large sizes at lower temperatures.

## 4. Discussion

### 4.1. Modelling Growth

The relationship between age and length for fish species, including those found in the Flemish Cap, has been extensively described using the VBGF [26,35,36,37]. However, the limitations of the VBGF parameters in incorporating environmental factors have been recognized, and alternative non-linear models such as generalised additive models have emerged to provide more flexible approaches [38]. This study demonstrates that GAMMs can provide improved explanations for length at age for multiple species by incorporating environmental variables, namely, bottom temperature in this case. The inclusion of bottom temperature provides the possibility to predict length at age under different environmental conditions, predictions which can then be used to fit traditional VBGF models to obtain comprehensive growth parameters. Some caution is, however, needed, as GAMMs provide purely empirical fits to the data, making no assumptions or including no information about the mechanisms underlying growth.

To validate the VBGFs which were derived from the GAMMs in this study, we can compare the growth parameters to results from previous investigations. The ranges of *L_∞_* and *k* for all species–sex combinations in this study were close to previous VBGF parameter estimates in the same area or the nearby NAFO divisions, with the differences being typically within 10% (see [26,27,28,29,39,40]). In the cases where multiple estimations were reported throughout the years such as for American plaice and roughhead grenadier, those estimations overlapped with the range found in this study [27,28]. This suggests that the approach used in this study provides biologically realistic estimations while giving additional insights into the temperature effects on growth. The largest difference we found was for female Greenland halibut, for which the recent literature has estimated *L_∞_* to be 109 cm, and *k* to be 0.09 year^−1^ [29]. However, the samples were obtained from Northeast Newfoundland and Labrador Shelves (NAFO SA2 + 3K) in 1976 and 1977, as well as from individuals smaller than 21 cm collected in 2007, which could be the source of the discrepancy. Currently, there is no recent VBGF parameter estimation for Greenland halibut from Flemish Cap that could permit a more direct comparison.

Although the models yielded estimates within apparently realistic ranges, there are some limitations. The modelling approach in this study took into account only the local temperature at the captured age and not the variation that an individual could have experienced throughout its life. Therefore, the results did not capture the variability of growth due to temperature fluctuation in an individual lifetime. We explored the possibility of cooperating temperatures from a previous year at the same location in modelling, but the results showed no improvement to the GAMM fitness. Still, the result from a previous investigation using the individual Atlantic cod otolith increment as a growth index found a similar increase in growth with temperature, especially for juvenile and young mature fish in the pre-moratorium period [5]. However, there is no study of otolith increments in relation to temperature for other species from Flemish Cap. The models constructed in this study provide a simple representation of the temperature effects on growth, but there are other factors that could be influential, such as fishing mortality, the thickness of the cold intermediate water layer, food availability and competition, that were not included in the analysis. This could be remedied in the future with the inclusion of other factors in the models.

Another limitation that should be taken into account is the unevenness of sampling for fish of different ages. This is particularly apparent at early ages for redfish, due to the impossibility of identifying the vast majority of juveniles to the species level. The exclusion of juvenile redfish up to 15 cm FL could obscure the growth trajectory in early life and the temperature impacts on it for all three redfish species. The resulting lack of samples for small individuals may have contributed to the larger size at age 0 in the models than was seen for other species. Additionally, catchability in the trawl nets used for sampling is typically lower for small individuals of all species [41]. The catchability of cod by the surveys used in this study was found to be very low at the age of 1 year and rapidly increase to a stable level at the age of 4 years [42]. On the other side of the spectrum, the inherently low number of old individuals in the population structure due to fishery and natural mortality resulted in the low number of samples representing the late life stage [43]. The last few age classes for each species (for both sexes) in this study were represented by fewer than ten samples, which may add to the uncertainty in VBGF parameter estimation [44].

### 4.2. Maximum Age and Size Variation

Beside their potential effects on the relationship between age and length, some of the external factors that were not accounted for in the present study clearly do affect the maximum age and size of multiple species, certainly in the case of fishing pressure. Clear trends of increasing maximum age and size from the mid-1990s to the mid-2010s were found for Atlantic cod and American plaice. This was likely a result of the reduced fishing pressure following the drastic depletion of the cod stock between the late 1980s and the early 1990s. Cod fishing mortality peaked between 1992 and 1995, before dropping sharply, and the moratorium was eventually implemented in 1999 [42]. This lifting of the fishing pressure would have allowed more individuals to grow older and reach larger size without being eliminated by fisheries. The maximum age and size increased steadily until the early 2010s, when the stock was deemed to have recovered, and fishing mortality was introduced once again but at a much lower rate compared to that in the pre-moratorium era. Although American plaice was generally not reported as the main target species in NAFO Division 3M [45], the great reduction in cod trawling activities may have positively impacted their population. At Flemish Cap, American plaice generally inhabit the shallow parts of the underwater mountain, similar to cod [33]. The easing of the fishing pressure on cod would have resulted in a reduction in American plaice bycatches. This was evident in the decline in fishing mortality for American plaice in parallel with the decline in fishing mortality for cod [46]. These changes in the maximum age and size resulting from changes in the fishing pressure could also have played a role in the growth trajectory variation, and further investigation would be needed to distinguish any such effects from the temperature effects.

Another species that showed a clear temporal trend in maximum age and length is beaked redfish. Beaked redfish maximum age and length declined throughout the mid-1990s to the late 2000s, even though redfish fishing mortality dropped sharply in 1997 and was subsequently kept at a low level [47]. This opposing temporal trend (compared to that for cod) could be explained by their prey–predator relationship [48]. The positive effect of reducing the fishing pressure on beaked redfish in the late 1990s could be negated by the increase in the predation pressure from Atlantic cod, which started to recover their spawning stock biomass in the early 2000s [41]. The growing number of older and larger cod would have consumed more and larger beaked redfish.

For Acadian and golden redfish, which are also eaten by Atlantic cod [21], the lack of otolith readings in multiple years (9 years for Acadian redfish, and 12 years for golden redfish) made it difficult to determine if there was a temporal trend for maximum age and length as well as to detect the effects of temperature on growth. Nevertheless, the data still covered the temperature range commonly seen in the area, and the GAMMs were still able to detect the effects of temperature on growth.

The geographical coverage of the first eleven years of the study period (1993–2003) would have resulted in deeper living populations being less well sampled, particularly those of roughhead grenadier and adult Greenland halibut, which are known to have a distribution range extending much deeper than 730 m [22,45]. The underrepresentation of older and larger Greenland halibut and roughhead grenadier in the earlier years became apparent when the age of the samples from 2004 onwards was determined. Only when the depth range of trawl sampling expanded deeper, reaching 1460 m, were the true maximum ages and sizes revealed. Moreover, the temperature at depths > 730 m is typically lower than in shallower waters, as shown by temperature maps [25], which is thus another source of bias in the data.

### 4.3. Temperature Effects on Fish

While the deep sea (>200 m depth) is generally considered more stable than shallow environments, it is not immune to oceanographical changes [49,50]. Most species show a decrease in *L_∞_* as the temperature rises, indicating a smaller size at later life stages. This response can be attributed to the dependence of ectothermic organisms on external heat sources for thermoregulation, which affects their metabolic rate [8]. Laboratory studies have demonstrated that increasing the temperature can accelerate fish metabolism [51,52]. Such changes in metabolic rate can subsequently influence other biological processes, including accelerating the early growth and reducing the maximum length, as observed in laboratory settings and natural habitats for the species examined in this study [53,54,55,56,57,58].

The results for some species challenge the assumption that warmer waters will lead to faster growth and smaller size. Male Greenland halibut, male Acadian redfish and female golden redfish displayed opposite trends. In the case of golden redfish, the changes were extremely small and might not be biologically significant, despite being statistically significant. The changes were more visible in male Greenland halibut and male Acadian redfish, but it should also be noted that large parts of their GAMM prediction 95% CIs overlapped at different temperatures, after 8 years of age for Greenland halibut and after age 12 years for Acadian redfish. It is important to consider the limited range of bottom temperatures experienced by deep-sea fish, with Greenland halibut distributed considerably deeper than other species [22]. The warmest temperature at great depths may not exceed their optimal threshold, unlike at the upper levels [59]. Studies in the nearby Gulf of St. Lawrence have shown that the optimum temperature for the metabolism and growth of Greenland halibut is exceeded at 7.5 °C, which is higher than the warmest recorded bottom temperature in the entire Flemish Cap [57]. Although the distribution of Acadian redfish is limited at a much shallower area, the increased temperature might still be within their optimal temperature range. This remains to be further investigated.

The impact of temperature on growth among demersal species can vary greatly, as evidenced by the trajectories of the fitted VBGFs. Some species, such as beaked redfish, exhibited clear changes in growth at different temperatures, while for others, such as American plaice, there was no significant variation. These diverse growth responses may be attributed to resilience to direct temperature impacts on biological processes and/or indirect effects such as changes in food accessibility [60,61]. For example, both fishery mortality and prey abundance (including redfish and northern shrimp abundance) are known to affect Atlantic cod growth in Flemish Cap, especially in their early life, and both can fluctuate over time, which could impact cod resilience to temperature changes [5]. Differences in habitat preference could also play a role. The closely taxonomically related *Sebastes* spp. show great variation in the growth responses to temperature, regardless of the shared similarity in feeding habits [21]. Beaked redfish is known to reside deeper than the other two redfish species [22], and the temperature changes they experience may thus affect their food sources (or competition for them) differently. The indirect temperature impacts through trophic interactions should be further evaluated for a more complete picture.

### 4.4. Ecological and Fishery Implications

The results generally show a potentially concerning trend of decreasing *L_∞_* and increasing *k* for multiple species as the bottom temperature rises above the average of 3.6 °C in Flemish Cap. A higher *k* is associated with a faster growth rate but often at the cost of a shorter lifespan, as observed for multiple species in nature and controlled environments [6,17,62]. Such trends suggest that fish populations may become shorter lived and have a higher natural mortality, which is especially concerning for species that must withstand a high fishing mortality [63]. Among the species examined in this investigation, Atlantic cod exhibited the most drastic shift towards a higher *k*. It is also important to note that the same maximum ages were shared for maximum predicted length in all scenarios, and the temperature effect on maximum age was not assessed in our study. In the case of Atlantic cod, this implied a higher maximum length (at the maximum age of 12 years) at higher temperatures for males due to the drastically increase in *k* (75% increase from 3 to 5 °C) even though *L_∞_* declined (17% decrease from 3 to 5 °C). For female cod, the maximum sizes (at the maximum age of 13 years) were only slightly lower at higher temperatures. However, this may not hold true if their longevity declines. If higher *k* were to reduce their longevity, their maximum size could also be further reduced [41,64].

Changes in the biological characteristics of species due to rising temperature can pose a threat to their populations and overall ecosystem dynamics [65]. Increased natural mortality has already been detected for Acadian and beaked redfish in Flemish Cap, despite the low fishing mortality; the increase in the individual growth rate of Atlantic cod was suspected to be a cause [66]. Warmer temperatures also lead to modifications in competition, with cold-preference species struggling to compete [67]. While the competitiveness of Atlantic cod may increase in the short term as a result of the faster early growth rate, the population could suffer a reduction in individual size and productivity. A meta-analysis of multiple fish species has shown that large females are disproportionately more fecund than smaller females, which potentially means a reduction in productivity if the individuals in the population become smaller [68]. In such a scenario, more resilient competitors that suffer less change such as Greenland halibut, which rely on similar food resources [21], may fare better in the long term. Changes in the life history of their major prey species, *Sebastes* spp., may also impact food availability for cod and further complicate the ecological dynamics [69]. Due to the complicated and unequal impacts of temperature on different species, a close monitoring of the ecosystem, particularly cod growth and its predation pressure on other species, is recommended in preparation for timely management responses.

The alteration of fish growth and natural mortality can have multiple implications on fisheries [70]. A decrease in length at age can result in the capture of smaller fish, which tend to be less valuable in the market, as demonstrated for Atlantic cod [71]. A smaller fish size, even with the same numerical abundance, would also reduce the amount of exploitable biomass that can be sustainably harvested [56]. Furthermore, the potential reduction in fecundity due to the decline in female size could result in lower productivity to sustain fisheries [68]. Additionally, the potential decrease in lifespan and increase in natural mortality will further limit the sustainable level of fishing mortality that can be imposed on the population [72]. Given that some commercially valuable species included in this study exhibit these trends, the severity of the decline in resource availability for fisheries needs to be investigated.

It is crucial to incorporate environmental effects into stock assessments, as indicated by this study and numerous others that highlight the influence of environmental conditions, particularly temperature, on fish life history [73]. Recent years have shown advancements in incorporating environmental factors into stock assessments and ecosystem modelling through models such as end-to-end OSMOSE and Atlantis [74,75]. However, many of these models primarily utilize environmental factors to forecast primary productivity, recruitment or distribution through coupled oceanographic models [76,77]. Exploring climate change impacts through studies of changes in individual life history is still rare [78]. The results of this study suggest that it could be valuable to further develop ecological models for Flemish Cap, such as the already existing multispecies Gadget model (GadCap), which relies on von Bertalanffy growth parameters to simulate the ecosystem in various temperature scenarios [36,79,80]. The predicted growth parameters, alongside other factors that could be altered by temperature, could be used as inputs in ecological models to simulate the ecosystem in various temperature scenarios.

## 5. Conclusions

The changes in the growth parameters of the species found through the modelling approach applied in the present study reinforce the evidence and concerns that the rising ocean temperature can result in undesirable alterations to fish growth, with likely knock-on effects at the ecosystem level, for example, mediated by changing trophic relationships linked to changes in body size. The magnitude of the temperature impact may vary among species. The mechanisms could involve their inherent biological characteristics or indirect impacts via their food sources and competition, which needs to be further investigated. Other factors that could affect growth and maximum body size, including fishing mortality, should be further explored. It is advisable to include the temperature effects on growth in assessments and ecosystem modelling for more comprehensive predictions that reflect the natural reality. The ocean temperature will not stop warming in the foreseeable future, and the race against time to figure out its impacts on the ecosystem and resources is needed to be taken seriously for us to prepare for the changes to come.

## Figures and Tables

**Table 1:** Number of samples whose age was determined through otolith reading from 1993 to 2018.

Year	Atlantic Cod		American Plaice		Greenland Halibut		Roughhead Grenadier		Acadian Redfish		Beaked Redfish		Golden Redfish	
	Female	Male	Female	Male	Female	Male	Female	Male	Female	Male	Female	Male	Female	Male
1993	917	896	533	459	465	391	-	-	239	242	185	225	176	201
1994	866	759	579	428	492	364	121	109	288	316	362	370	305	318
1995	703	768	396	345	356	244	260	224	398	346	344	341	381	454
1996	838	698	232	302	502	355	169	221	448	425	606	661	508	549
1997	762	569	211	210	694	568	-	-	49	78	243	279	150	121
1998	278	176	247	183	1040	875	304	289	182	196	257	234	-	-
1999	154	126	297	143	454	358	288	267	459	486	458	519	314	368
2000	131	111	184	190	634	524	304	296	37	38	232	233	105	75
2001	251	207	200	339	426	397	436	331	-	-	94	90	-	-
2002	162	139	155	67	447	400	179	146	117	102	181	171	58	71
2003	152	119	162	358	425	379	270	239	-	-	131	119	-	-
2004	399	277	-	-	541	449	-	-	-	-	-	-	-	-
2005	329	305	169	162	620	435	318	200	-	-	128	132	-	-
2006	534	505	116	183	635	487	191	121	-	-	69	80	-	-
2007	686	657	82	102	593	446	192	105	-	-	83	78	-	-
2008	802	839	204	201	569	397	183	118	-	-	-	-	-	-
2009	595	590	196	216	619	377	200	113	170	107	170	118	-	-
2010	532	430	187	204	479	350	525	343	-	-	-	-	-	-
2011	788	671	310	214	694	484	594	478	187	162	182	167	-	-
2012	698	592	358	377	455	253	387	278	276	153	282	164	339	174
2013	861	741	343	316	421	291	366	296	404	150	344	157	439	1
2014	416	320	-	-	77	51	144	107	112	37	85	66	161	1
2015	-	-	-	-	-	-	-	-	-	-	-	-	-	-
2016	793	616	461	469	408	257	379	300	152	111	166	99	376	123
2017	392	311	-	-	40	26	129	81	68	49	32	40	42	46
2018	211	196	-	-	87	43	127	88	43	34	46	47	68	10
Total	13,250	11,618	5622	5468	12,173	9201	6066	4750	3629	3032	4680	4390	3422	2512

**Table 2:** Maximum age (*t_max_*) of seven demersal fish species by sex from otolith reading from 1993 to 2018.

Species	Sex	*t_max_* ± SE (years)
Atlantic cod	All	13.3 ± 0.2
	Female	13.3 ± 0.2
	Male	11.5 ± 0.2
American plaice	All	25.6 ± 0.3
	Female	25.5 ± 0.3
	Male	22.6 ± 0.5
Greenland halibut	All	19.5 ± 0.4
	Female	19.5 ± 0.4
	Male	12.6 ± 0.2
Roughhead grenadier	All	26.4 ± 0.7
	Female	26.4 ± 0.7
	Male	18.5 ± 0.5
Acadian redfish	All	24.6 ± 0.9
	Female	24.6 ± 0.9
	male	19.7 ± 0.5
Beaked redfish	All	35.6 ± 1.1
	Female	29.7 ± 1.0
	Male	35.0 ± 1.2
Golden redfish	All	33.9 ± 1.3
	Female	32.0 ± 1.3
	Male	30.2 ± 1.2

**Table 3:** Relative goodness of fit for candidate length-at-age models for females and males of all species, comparing models which (i) included the effects of bottom temperature (BT) and its interaction with age, (ii) included BT only as a main effect and (iii) did not include BT. For each species and sex combination, the models are listed in order of goodness of fit, the best model being listed first. The table displays effective degree of freedom (edf), deviance explained (DE) and differences in AIC between the best-fit models and other candidate models (ΔAIC).

Species	Female				Male			
	Model	edf	DE (%)	ΔAIC	Model	edf	DE (%)	ΔAIC
Atlantic cod	*s*(*Age, BT*)	27.12	87.9		*s*(*Age, BT*)	26.09	86.4	
	*s*(*Age*) + s(*BT*)	12.70	87.8	454.00	*s*(*Age*) + s(*BT*)	13.34	86.3	419.27
	*s*(*Age*)	7.97	86.8	547.70	*s*(*Age*)	7.97	85.1	522.57
American plaice	*s*(*Age*)	8.43	90.4		*s*(*Age*)	8.36	80.6	
	*s*(*Age*) + s(*BT*)	10.33	90.4	3.60	*s*(*Age*) + s(*BT*)	9.79	80.6	7.60
	*s*(*Age*, *BT*)	25.94	90.4	18.21	*s*(*Age*, *BT*)	25.93	80.7	21.44
Greenland halibut	*s*(*Age, BT*)	26.22	94.1		*s*(*Age, BT*)	24.05	90.7	
	*s*(*Age*) + s(*BT*)	13.70	94.1	106.53	*s*(*Age*) + s(*BT*)	13.95	90.7	13.67
	*s*(*Age*)	8.3	94.0	161.02	*s*(*Age*)	8.54	90.6	49.38
Roughhead grenadier	*s*(*Age*) + s(*BT*)	11.52	96.0		*s*(*Age*) + s(*BT*)	11.48	91.6	
	*s*(*Age*)	8.26	96.0	6.56	*s*(*Age*)	7.26	91.3	4.31
	*s*(*Age, BT*)	24.44	96.0	60.96	*s*(*Age, BT*)	23.67	91.6	26.85
Acadian redfish	*s*(*Age, BT*)	21.93	91.0		*s*(*Age, BT*)	22.23	86.8	
	*s*(*Age*) + s(*BT*)	11.01	90.9	30.09	*s*(*Age*) + s(*BT*)	8.42	86.3	54.10
	*s*(*Age*)	8.12	90.7	33.64	*s*(*Age*)	7.41	86.0	57.47
Beaked redfish	*s*(*Age, BT*)	24.97	93.3		*s*(*Age, BT*)	25.25	91.5	
	*s*(*Age*) + s(*BT*)	10.91	93.1	86.35	*s*(*Age*) + s(*BT*)	8.71	90.9	144.51
	*s*(*Age*)	7.67	92.8	111.62	*s*(*Age*)	7.72	90.3	179.89
Golden redfish	*s*(*Age, BT*)	21.07	92.2		*s*(*Age, BT*)	21.61	94.8	
	*s*(*Age*)	7.10	92.1	13.39	*s*(*Age*)	7.89	94.7	3.81
	*s*(*Age*) + s(*BT*)	8.10	92.1	20.66	*s*(*Age*) + s(*BT*)	8.91	94.7	10.37

**Table 4:** Growth parameters derived from fitting von Bertalanffy growth functions to GAMM predictions in three bottom temperature (BT) scenarios.

Species	BT (°C)	Female			Male		
		*L_∞_* (cm)	*k* (Year^−1^)	*t*_0_ (Years)	*L_∞_* (cm)	*k* (Year^−1^)	*t*_0_ (Years)
Atlantic cod	3	234.53	0.05	−0.63	158.78	0.08	−0.55
	3.5	205.31	0.07	−0.5	147.79	0.1	−0.44
	4	167.02	0.09	−0.29	141.84	0.11	−0.31
	4.5	158.64	0.12	−0.24	132.64	0.13	−0.24
	5	139.74	0.13	−0.33	132.12	0.14	−0.3
American plaice	NA	54.22	0.21	−0.37	43.09	0.27	−0.47
Greenland halibut	3	173.02	0.03	−2.26	87.42	0.09	−1.33
	3.5	150.62	0.04	−2.11	86.37	0.1	−1.22
	4	135.05	0.05	−1.85	92.59	0.09	−1.19
	4.5	126.57	0.06	−1.56	101.69	0.08	−1.19
	5	121.8	0.06	−1.31	110.69	0.07	−1.21
Roughhead grenadier	3	61.66	0.04	−0.53	26.59	0.13	−0.24
	3.5	61.2	0.04	−0.34	25.87	0.13	−0.02
	4	61.18	0.04	−0.33	25.87	0.13	−0.02
	4.5	61.32	0.04	−0.39	25.8	0.13	0.01
	5	61.42	0.04	−0.43	25.62	0.13	0.06
Acadian redfish	3	38.75	0.11	−2.15	29.99	0.2	−1.43
	3.5	38.2	0.12	−2.17	30.28	0.19	−1.52
	4	37.66	0.12	−2.26	30.65	0.18	−1.71
	4.5	37.14	0.12	−2.38	31.07	0.16	−1.98
	5	36.65	0.12	−2.51	31.52	0.15	−2.27
Beaked redfish	3	46.06	0.08	−2.87	41.23	0.11	−2
	3.5	43.9	0.09	−2.86	39.46	0.11	−2.12
	4	41.77	0.09	−2.88	37.65	0.11	−2.32
	4.5	39.76	0.1	−2.89	35.85	0.11	−2.53
	5	37.91	0.1	−2.89	34.09	0.12	−2.7
Golden redfish	3	59.25	0.06	−2.31	47	0.09	−1.83
	3.5	59.92	0.06	−2.43	46.08	0.09	−1.82
	4	60.6	0.06	−2.56	45.24	0.1	−1.82
	4.5	61.17	0.06	−2.67	44.51	0.1	−1.83
	5	61.65	0.06	−2.79	43.89	0.1	−1.86

## Data Availability

The survey data underlying this article belong to the Institute of Marine Research—the Spanish National Research Council (IIM-CSIC). The data will be shared upon request to the corresponding author, subjected to permission of the IIM-CSIC.

## Supplementary Materials

The following supporting information can be downloaded at: https://www.mdpi.com/article/10.3390/ani15081120/s1, Figure S1: GAMM surface plots of length in relation to age and bottom temperature. Note that the scale of the x, y and z-axes varies among plots for display purposes.

## References

[1] Abraham J.P., Baringer M., Bindoff N.L., Boyer T., Cheng L.J., Church J.A., Conroy J.L., Domingues C.M., Fasullo J.T., Gilson J. (2013). A Review of Global Ocean Temperature Observations: Implications for Ocean Heat Content Estimates and Climate Change. Rev. Geophys..

[2] Saba V.S., Griffies S.M., Anderson W.G., Winton M., Alexander M.A., Delworth T.L., Hare J.A., Harrison M.J., Rosati A., Vecchi G.A. (2016). Enhanced Warming of the Northwest Atlantic Ocean under Climate Change. J. Geophys. Res. Ocean..

[3] Alexander M.A., Shin S.I., Scott J.D., Curchitser E., Stock C. (2020). The Response of the Northwest Atlantic Ocean to Climate Change. J. Clim..

[4] Swain D.P., Benoît H.P. (2015). Extreme Increases in Natural Mortality Prevent Recovery of Collapsed Fish Populations in a Northwest Atlantic Ecosystem. Mar. Ecol. Prog. Ser..

[5] Ruiz-Díaz R., Dominguez-Petit R., Saborido-Rey F. (2022). Atlantic Cod Growth History in Flemish Cap Between 1981 and 2016: The Impact of Fishing and Climate on Growth Performance. Front. Mar. Sci..

[6] Gislason H., Daan N., Rice J.C., Pope J.G. (2010). Size, Growth, Temperature and the Natural Mortality of Marine Fish. Fish Fish..

[7] Killen S.S. (2014). Growth Trajectory Influences Temperature Preference in Fish through an Effect on Metabolic Rate. J. Anim. Ecol..

[8] Schulte P.M. (2015). The Effects of Temperature on Aerobic Metabolism: Towards a Mechanistic Understanding of the Responses of Ectotherms to a Changing Environment. J. Exp. Biol..

[9] Crozier L.G., Hutchings J.A. (2014). Plastic and Evolutionary Responses to Climate Change in Fish. Evol. Appl..

[10] Tanner S.E., Giacomello E., Menezes G.M., Mirasole A., Neves J., Sequeira V., Vasconcelos R.P., Vieira A.R., Morrongiello J.R. (2020). Marine Regime Shifts Impact Synchrony of Deep-Sea Fish Growth in the Northeast Atlantic. Oikos.

[11] Pörtner H.O., Peck M.A. (2010). Climate Change Effects on Fishes and Fisheries: Towards a Cause-and-Effect Understanding. J. Fish Biol..

[12] Doney S.C., Ruckelshaus M., Emmett Duffy J., Barry J.P., Chan F., English C.A., Galindo H.M., Grebmeier J.M., Hollowed A.B., Knowlton N. (2012). Climate Change Impacts on Marine Ecosystems. Ann. Rev. Mar. Sci..

[13] Cheung W.W.L., Frölicher T.L., Lam V.W.Y., Oyinlola M.A., Reygondeau G., Rashid Sumaila U., Tai T.C., Teh L.C.L., Wabnitz C.C.C. (2021). Marine High Temperature Extremes Amplify the Impacts of Climate Change on Fish and Fisheries. Sci. Adv..

[14] Hughes T.P., Bellwood D.R., Folke C., Steneck R.S., Wilson J. (2005). New Paradigms for Supporting the Resilience of Marine Ecosystems. Trends Ecol. Evol..

[15] Huang M., Ding L., Wang J., Ding C., Tao J. (2021). The Impacts of Climate Change on Fish Growth: A Summary of Conducted Studies and Current Knowledge. Ecol. Indic..

[16] Sparholt H., Bogstad B., Christensen V., Collie J., Van Gemert R., Hilborn R., Horbowy J., Howell D., Melnychuk M.C., Pedersen S.A. (2021). Estimating Fmsy from an Ensemble of Data Sources to Account for Density Dependence in Northeast Atlantic Fish Stocks. ICES J. Mar. Sci..

[17] Lee W.S., Monaghan P., Metcalfe N.B. (2013). Experimental Demonstration of the Growth Rate-Lifespan Trade-Off. Proc. R. Soc. B Biol. Sci..

[18] Folkvord A., Jørgensen C., Korsbrekke K., Nash R.D.M., Skjæraasen J.E., Nilsen T. (2014). Trade-Offs between Growth and Reproduction in Wild Atlantic Cod. Can. J. Fish. Aquat. Sci..

[19] Pérez-Rodríguez A., Morgan J., Koen-Alonso M., Saborido-Rey F. (2013). Disentangling Genetic Change from Phenotypic Response in Reproductive Parameters of Flemish Cap Cod *Gadus morhua*. Fish. Res..

[20] Devine B.M., Baker K.D., Edinger E.N., Fisher J.A.D. (2020). Habitat Associations and Assemblage Structure of Demersal Deep-Sea Fishes on the Eastern Flemish Cap and Orphan Seamount. Deep. Sea Res. 1 Oceanogr. Res. Pap..

[21] Pérez-Rodríguez A., Koen-Alonso M., González-Iglesias C., Saborido-Rey F. (2011). Analysis of Common Trends in the Feeding Habits of Main Demersal Fish Species on the Flemish Cap.

[22] Nogueira A., Paz X., González-Troncoso D. (2017). Demersal Groundfish Assemblages and Depth-Related Trends on Flemish Cap (NAFO Division 3M): 2004–2013. Fish. Res..

[23] Höffle H., Planque B. (2023). Natural Mortality Estimations for Beaked Redfish (Sebastes Mentella)—A Long-Lived Ovoviviparous Species of the Northeast Arctic. Fish. Res..

[24] Colbourne E., Perez-Rodriguez A., Cabrero A., Gonzalez_Nuevo G. (2018). Ocean Climate Variability on the Flemish Cap in NAFO Subdivision 3M During 2017.

[25] Drévillon M., Lellouche J.-M., Régnier C., Garric G., Bricaud C., Hernandez O., Bourdallé-Badie R. (2021). Quality Information Document for Global Ocean Reanalysis Products: GLOBAL_REANALYSIS_PHY_001_030.

[26] Saborido-Rey F., Garabana D., Cerviño S. (2004). Age and Growth of Redfish (Sebastes Marinus, S. Mentella, and S. Fasciatus) on the Flemish Cap (Northwest Atlantic). ICES J. Mar. Sci..

[27] Murua H., Gónzalez F. (2007). A Review on Roughhead Grenadier (Macrourus Berglax) Biology and Population Structure on Flemish Cap (NAFO Division 3M) 1991-2006 Based upon EU Flemish Cap Bottom Survey Data.

[28] González-Troncoso D., Paz X. (2008). Growth Features of American Plaice Hippoglossoides Platessoides in Northwest Atlantic.

[29] Dwyer K.S., Treble M.A., Campana S.E. (2016). Age and Growth of Greenland Halibut (Reinhardtius Hippoglossoides) in the Northwest Atlantic: A Changing Perception Based on Bomb Radiocarbon Analyses. Fish. Res..

[30] Vázquez A., Miguel Casas J., Alpoim R. (2014). Protocols of the EU Bottom Trawl Survey of Flemish Cap. NAFO Sci. Counc. Stud..

[31] NAFO (2023). Northwest Atlantic Fisheries Organization Conservation and Enforcement Measures 2023.

[32] González-Troncoso D., Rábade S., Casas Sánchez J.M., Garrido I., Fabeiro M., Román E., Alpoim R. (2023). Results from Bottom Trawl Survey on Flemish Cap of June–July 2022.

[33] Songin K., Pierce G., Saborido-Rey F. (2024). Spatiotemporal Changes in the Atlantic Cod (*Gadus morhua*) Stock at Flemish Cap (1993–2019) and Their Relationships with Demersal Communities. J. Northwest Atl. Fish. Sci..

[34] Wood S.N. (2023). Mgcv: Mixed GAM Computation Vehicle with Automatic Smoothness Estimation. Generalized Additive Models: An Introduction with R.

[35] Chen Y., Jackson D.A., Harvey H.H. (1992). A Comparison of von Bertalanffy and Polynomial Functions in Modelling Fish Growth Data. Can. J. Fish. Aquat. Sci..

[36] Murua H. (2003). Population Structure, Growth and Reproduction of Roughhead Grenadier on the Flemish Cap and Flemish Pass. J. Fish Biol..

[37] Pérez-Rodríguez A., Howell D., Casas M., Saborido-Rey F., Ávila-De Melo A. (2017). Dynamic of the Flemish Cap Commercial Stocks: Use of a Gadget Multispecies Model to Determine the Relevance and Synergies among Predation, Recruitment, and Fishing. Can. J. Fish. Aquat. Sci..

[38] Kimura D.K. (2008). Extending the von Bertalanffy Growth Model Using Explanatory Variables. Can. J. Fish. Aquat. Sci..

[39] Brunel T. (2019). Investigation of a Growth Model Incorporating Density-Dependence for the Cod 3M Management Plan Simulations.

[40] Varkey D., Gullage N., Regular P.M., Kumar R. (2023). Review and Update of the State-Space Management Strategy Evaluation for Greenland Halibut in NAFO Subarea 2 and Divisions 3KLMNO with MseSurv.

[41] Fraser H.M., Greenstreet S.P.R., Piet G.J. (2007). Taking Account of Catchability in Groundfish Survey Trawls: Implications for Estimating Demersal Fish Biomass. ICES J. Mar. Sci..

[42] Garrido I., González-Troncoso D., González-Costas F. (2023). Assessment of the Cod Stock in NAFO Division 3M..

[43] Ohlberger J., Langangen Ø., Stige L.C. (2022). Age Structure Affects Population Productivity in an Exploited Fish Species. Ecol. Appl..

[44] Kritzer J.P., Davies C.R., Mapstone B.D. (2001). Characterizing Fish Populations: Effects of Sample Size and Population Structure on the Precision of Demographic Parameter Estimates. Can. J. Fish. Aquat. Sci..

[45] NAFO (2024). Annual Fisheries Statistics Databases STATLANT 21.

[46] Alpoim R. (2023). An Assessment of American Plaice (Hippoglossoides Platessoides) in NAFO Division 3M..

[47] Gonçalves P., Alpoim R., Ávila de Melo A. (2023). Redfish Div. 3M Biological Reference Points and Advice under the PA Alternative Framework.

[48] Iglesias G., González-Costas F., González-Troncoso D. (2012). Atlantic Cod Predation on Redfish in Flemish Cap.

[49] Meehl G.A., Arblaster J.M., Fasullo J.T., Hu A., Trenberth K.E. (2011). Model-Based Evidence of Deep-Ocean Heat Uptake during Surface-Temperature Hiatus Periods. Nat. Clim. Chang..

[50] Roberts E.M., Mienis F., Rapp H.T., Hanz U., Meyer H.K., Davies A.J. (2018). Oceanographic Setting and Short-Timescale Environmental Variability at an Arctic Seamount Sponge Ground. Deep. Sea Res. 1 Oceanogr. Res. Pap..

[51] Claireaux G., Webber D.M., Lagardère J.P., Kerr S.R. (2000). Influence of Water Temperature and Oxygenation on the Aerobic Metabolic Scope of Atlantic Cod (*Gadus morhua*). J. Sea Res..

[52] Tirsgaard B., Behrens J.W., Steffensen J.F. (2015). The Effect of Temperature and Body Size on Metabolic Scope of Activity in Juvenile Atlantic Cod *Gadus morhua* L.. Comp. Biochem. Physiol. A Mol. Integr. Physiol..

[53] Sünksen K., Stenberg C., Grønkjær P. (2010). Temperature Effects on Growth of Juvenile Greenland Halibut (Reinhardtius Hippoglossoides Walbaum) in West Greenland Waters. J. Sea Res..

[54] Baudron A.R., Needle C.L., Rijnsdorp A.D., Tara Marshall C. (2014). Warming Temperatures and Smaller Body Sizes: Synchronous Changes in Growth of North Sea Fishes. Glob. Chang. Biol..

[55] Tirsgaard B., Svendsen J.C., Steffensen J.F. (2015). Effects of Temperature on Specific Dynamic Action in Atlantic Cod *Gadus morhua*. Fish Physiol. Biochem..

[56] van Rijn I., Buba Y., DeLong J., Kiflawi M., Belmaker J. (2017). Large but Uneven Reduction in Fish Size across Species in Relation to Changing Sea Temperatures. Glob. Chang. Biol..

[57] Ghinter L., Lambert Y., Audet C. (2021). Juvenile Greenland Halibut (Reinhardtius Hippoglossoides) Growth in the Context of Rising Temperature in the Estuary and Gulf of St. Lawrence. Fish. Res..

[58] Martínez-Silva M.A., Vagner M., Senay C., Audet C. (2022). Using Gene Expression to Identify the Most Suitable Environmental Conditions for Growth and Metabolism of Juvenile Deepwater Redfish (Sebastes Mentella) in the Estuary and the Gulf of St. Lawrence. ICES J. Mar. Sci..

[59] Richter A., Kolmes S.A. (2005). Maximum Temperature Limits for Chinook, Coho, and Chum Salmon, and Steelhead Trout in the Pacific Northwest. Rev. Fish. Sci..

[60] Neuheimer A.B., Thresher R.E., Lyle J.M., Semmens J.M. (2011). Tolerance Limit for Fish Growth Exceeded by Warming Waters. Nat. Clim. Chang..

[61] O’Brien C.L., Spooner P.T., Wharton J.H., Papachristopoulou E., Dutton N., Fairman D., Garratt R., Li T., Pallottino F., Stringer F. (2021). Exceptional 20th Century Shifts in Deep-Sea Ecosystems Are Spatially Heterogeneous and Associated with Local Surface Ocean Variability. Front. Mar. Sci..

[62] Metcalfe N.B., Monaghan P. (2003). Growth versus Lifespan: Perspectives from Evolutionary Ecology. Exp. Gerontol..

[63] Rountrey A.N., Coulson P.G., Meeuwig J.J., Meekan M. (2014). Water Temperature and Fish Growth: Otoliths Predict Growth Patterns of a Marine Fish in a Changing Climate. Glob. Chang. Biol..

[64] Lilly G.R., Nakken O., Brattey J. (2013). A Review of the Contributions of Fisheries and Climate Variability to Contrasting Dynamics in Two Arcto-Boreal Atlantic Cod (*Gadus morhua*) Stocks: Persistent High Productivity in the Barents Sea and Collapse on the Newfoundland and Labrador Shelf. Prog. Oceanogr..

[65] Carozza D.A., Bianchi D., Galbraith E.D. (2019). Metabolic Impacts of Climate Change on Marine Ecosystems: Implications for Fish Communities and Fisheries. Glob. Ecol. Biogeogr..

[66] Ávila De Melo A., Saborido-Rey F., Troncoso D.G., Pochtar M., Alpoim R. (2011). An Assessment of Beaked Redfish (S. mentella and S. fasciatus) in NAFO Division 3M (With an Approach to the Likely Impact of Recent 3M Cod Growth on Redfish Natural Mortality).

[67] Urban M.C., Holt R.D., Gilman S.E., Tewksbury J. (2011). Heating up Relations between Cold Fish: Competition Modifies Responses to Climate Change. J. Anim. Ecol..

[68] Barneche D.R., Robertson D.R., White C.R., Marshall D.J. (2018). Fish Reproductive-Energy Output Increases Disproportionately with Body Size. Science.

[69] Devine J.A., Haedrich R.L. (2011). The Role of Environmental Conditions and Exploitation in Determining Dynamics of Redfish (Sebastes Species) in the Northwest Atlantic. Fish. Oceanogr..

[70] Barbeaux S.J., Holsman K., Zador S. (2020). Marine Heatwave Stress Test of Ecosystem-Based Fisheries Management in the Gulf of Alaska Pacific Cod Fishery. Front. Mar. Sci..

[71] Sogn-Grundvåg G., Zhang D., Henriksen E., Joensen S., Bendiksen B.I., Hermansen Ø. (2021). Fish Quality and Market Performance: The Case of the Coastal Fishery for Atlantic Cod in Norway. Mar. Policy.

[72] Szuwalski C.S., Hollowed A.B. (2016). Climate Change and Non-Stationary Population Processes in Fisheries Management. ICES J. Mar. Sci..

[73] Sydeman W.J., Poloczanska E., Reed T.E., Thompson S.A. (2015). Climate Change and Marine Vertebrates. Science.

[74] Fulton E.A., Link J.S., Kaplan I.C., Savina-Rolland M., Johnson P., Ainsworth C., Horne P., Gorton R., Gamble R.J., Smith A.D.M. (2011). Lessons in Modelling and Management of Marine Ecosystems: The Atlantis Experience. Fish Fish..

[75] Travers-Trolet M., Shin Y.J., Field J.G. (2014). An End-to-End Coupled Model ROMS-N2P2Z2D2-OSMOSE of the Southern Benguela Foodweb: Parameterisation, Calibration and Pattern-Oriented Validation. Afr. J. Mar. Sci..

[76] Fulton E.A. (2010). Approaches to End-to-End Ecosystem Models. J. Mar. Syst..

[77] Moullec F., Barrier N., Drira S., Guilhaumon F., Marsaleix P., Somot S., Ulses C., Velez L., Shin Y.J. (2019). An End-to-End Model Reveals Losers and Winners in a Warming Mediterranean Sea. Front. Mar. Sci..

[78] Travers-Trolet M., Bourdaud P., Genu M., Velez L., Vermard Y. (2020). The Risky Decrease of Fishing Reference Points Under Climate Change. Front. Mar. Sci..

[79] Christensen V., Walters C.J., Pauly D. (2005). Ecopath with Ecosim: A User’s Guide.

[80] Pérez-Rodríguez A., Umar I., Goto D., Howell D., Mosqueira I., González-Troncoso D. (2022). Evaluation of Harvest Control Rules for a Group of Interacting Commercial Stocks Using a Multispecies MSE Framework. Can. J. Fish. Aquat. Sci..

