# Supplementary material for: Bottom Temperature Effect on Growth of Multiple Demersal Fish Species in Flemish Cap, Northwest Atlantic
Source: Animals (Basel). 2025 Apr 12;15(8):1120. doi: 10.3390/ani15081120 (PMC12023976; doi:10.3390/ani15081120)
Supplement: Supplementary file 1 [file animals-15-01120-s001.zip › animals-3528269-supplementary.pdf]

Supplementary Materials

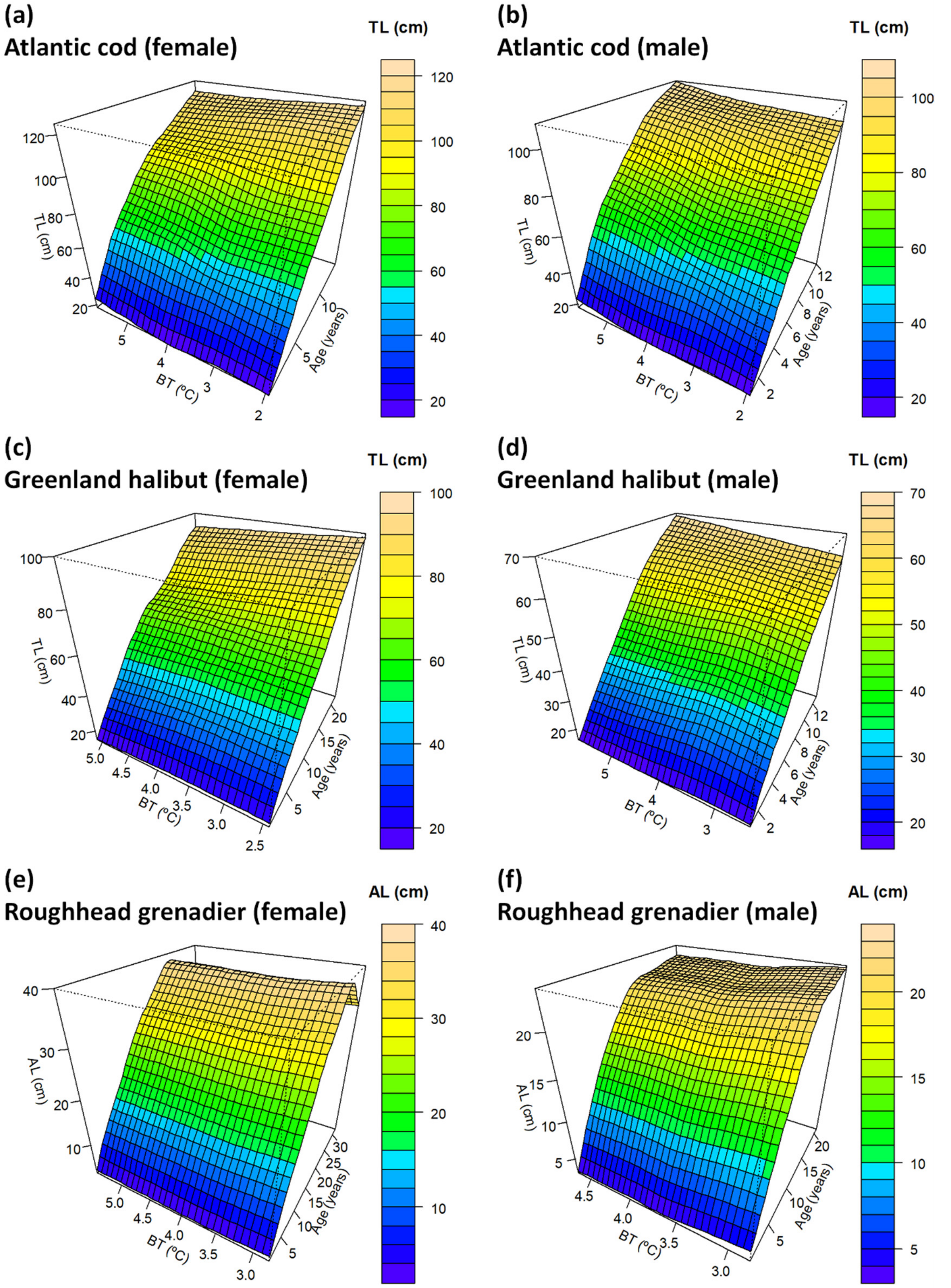

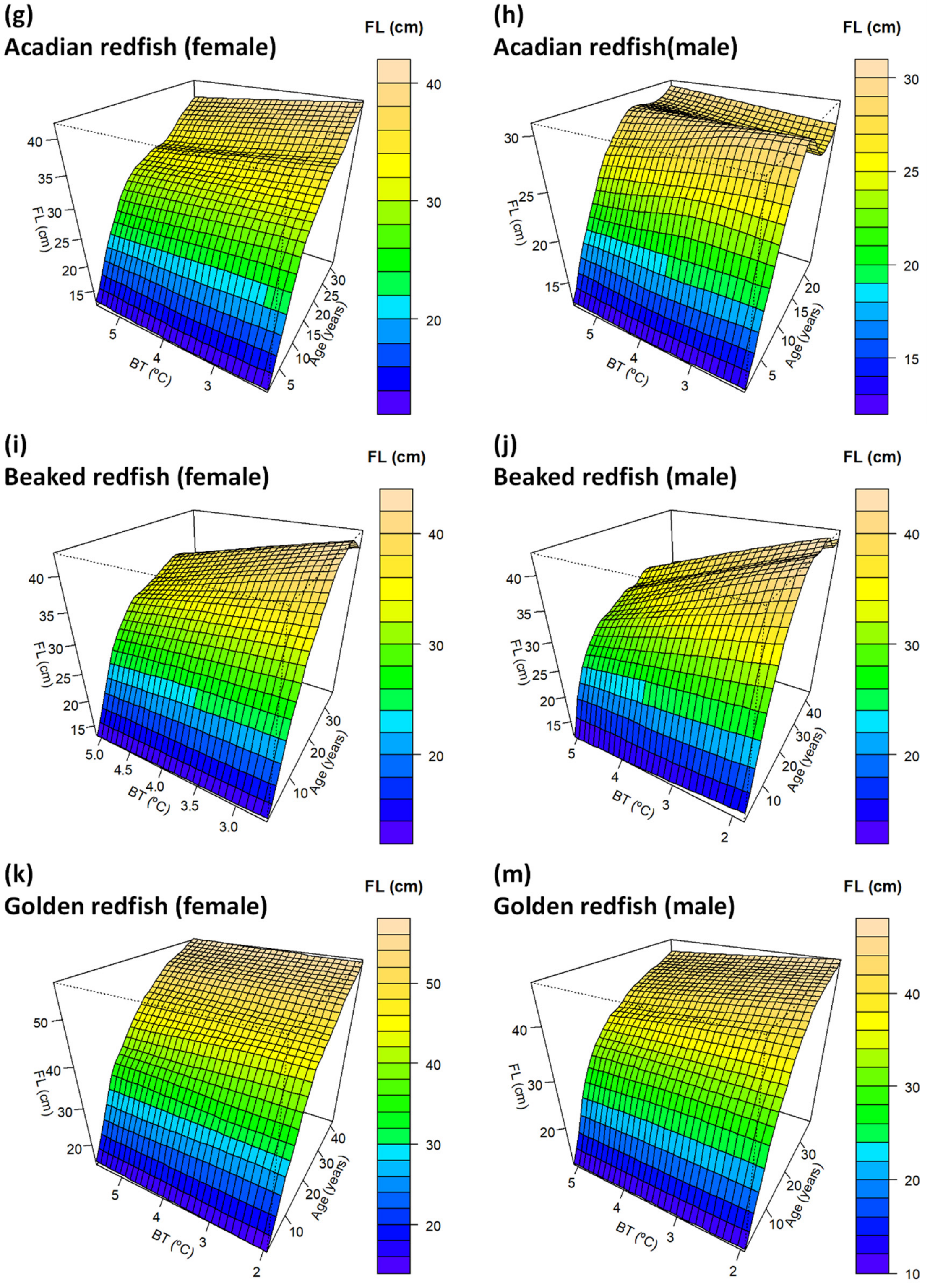

Figure S1. GAMM surface plots of length in relation to age and bottom temperature. Note that the scale of the x, y and z-axes varies among plots for display purposes.
